# Retinitis Pigmentosa: From Genetic Insights to Innovative Therapeutic Approaches—A Literature Review

**DOI:** 10.3390/medicina61071179

**Published:** 2025-06-29

**Authors:** Ricardo A. Murati Calderón, Andres Emanuelli, Natalio Izquierdo

**Affiliations:** 1 Department of Ophthalmology, School of Medicine, University of Puerto Rico, San Juan 00936-5067, Puerto Rico; aemanuelli@gmail.com; 2 Emanuelli Research and Development, Retina Care, Arecibo 00612-4368, Puerto Rico; 3 Department of Surgery, University of Puerto Rico, Medical Sciences Campus, San Juan 00936-5067, Puerto Rico; njuan@msn.com

**Keywords:** retinitis pigmentosa, genetics, inherited retinal disease, gene therapy, optogenetics, cell-based therapy, retinal prosthetics, multidisciplinary approach

## Abstract

Retinitis pigmentosa (RP) is a heterogeneous group of inherited retinal dystrophies characterized by progressive photoreceptor degeneration and vision loss. While current management is largely supportive—relying on visual aids, orientation training, and nutritional supplementation—these interventions offer only symptomatic relief and do not halt disease progression. Advances in molecular genetics have led to the development of targeted treatments, including gene replacement therapy, RNA-based therapies, and CRISPR/Cas9 gene editing, offering promising strategies for disease modification. The approval of voretigene neparvovec for *RPE65*-associated RP marked a milestone in gene therapy, while ongoing trials targeting mutations in *RPGR*, *USH2A*, and *CEP290* are expanding therapeutic options. Optogenetic therapy and stem cell transplantation represent additional strategies, particularly for patients with advanced disease. Challenges persist in delivery efficiency, immune responses, and treating large or dominant-negative mutations. Non-viral vectors, nanoparticle systems, and artificial intelligence-guided diagnostics are being explored to address these limitations and support personalized care. This review summarizes the current and emerging therapeutic landscape for RP, highlighting the shift toward precision medicine and the need for continued innovation to overcome genetic and phenotypic variability.

## 1. Introduction

### 1.1. Overview

Retinitis pigmentosa (RP) is a group of inherited retinal dystrophies characterized by the progressive degeneration of photoreceptor cells, ultimately leading to vision loss. Patients typically experience nyctalopia initially, followed by gradual peripheral vision constriction, and in advanced stages, progress to central vision loss [1]. The prevalence of RP is estimated at approximately 1 in 4000 individuals globally [2]. Most cases are classified as non-syndromic, where retinal degeneration occurs in isolation. However, approximately 30% of RP cases are syndromic, which means that they occur in conjunction with systemic features as part of broader genetic disorders such as the Usher syndrome and Bardet–Biedl syndrome [3].

Genetically, RP is highly heterogeneous, with mutations identified in over 80 genes inherited in autosomal dominant, autosomal recessive, or X-linked patterns [1]. Mutations in genes such as *RHO* (Rhodopsin)*, RP1*, and *RPGR* (retinitis pigmentosa GTPase regulator) have been among the most described, leading to a disruption in key photoreceptor functions with subsequent retinal degeneration [4,5]. *RHO* mutations are a leading cause of autosomal dominant RP (adRP), whereas *USH2A* (Usher Syndrome Type 2A) is the most frequently mutated gene in autosomal recessive RP (arRP) [6]. In contrast, mutations in *RPGR* account for most X-linked RP cases [7]. Due to the irreversible nature of photoreceptor loss, early diagnosis is crucial for optimizing disease management, allowing genetic counseling, lifestyle modifications, and the consideration of emerging therapeutic interventions to preserve vision [8]. Early disease identification is critical as many experimental therapies are mutation-specific and most effective when initiated before significant retinal damage has occurred.

### 1.2. Current Treatment Landscape

However, current treatment options for RP remain limited and are primarily supportive. These include low vision aids, orientation and mobility training, and nutritional supplements [9,10]. Low vision aids and orientation and mobility training help maximize residual vision and improve quality of life in patients with RP [11]. Nutritional supplements, particularly vitamin A palmitate, have been studied for their potential to slow disease progression, although the evidence remains mixed [12]. Device-based approaches, such as retinal prostheses, have also been developed to partially restore visual perception in patients with advanced RP, though accessibility and functional outcomes remain variable. Despite these interventions, existing therapies primarily address symptoms rather than the underlying genetic causes of RP. This limitation highlights the need for advanced therapeutic approaches, such as gene- and cell-based therapies, which target disease mechanisms and offer long-term solutions [13].

Recent years have witnessed transformative progress in RP therapeutics. The approval of voretigene neparvovec-rzyl (Luxturna^®^) for *RPE65*-associated retinal dystrophy marked a milestone in gene therapy, while ongoing clinical trials now explore gene editing (CRISPR), RNA-based correction strategies, optogenetics, and stem cell transplantation. Furthermore, the integration of artificial intelligence into molecular diagnostics and genotype–phenotype prediction is rapidly enhancing personalized care.

This review aims to synthesize the current understanding of retinitis pigmentosa from a genetic and molecular perspective while outlining the latest advancements in gene therapy, RNA-based interventions, optogenetics, and regenerative medicine. By examining the strengths and limitations of emerging therapeutic strategies, this work highlights the growing role of personalized, mutation-specific approaches in reshaping the management of RP.

## 2. Pathophysiology

### 2.1. Genetic Mutations

RP is caused by pathogenic mutations that impair critical photoreceptor cell functions. To date, more than 80 causative genes have been identified, many of which encode proteins involved in phototransduction, ciliary transport, protein folding, and visual cycle metabolism [8,14]. These include *RHO*, *RPGR*, *USH2A*, *RPE65*, and *PRPF31*, among others. Recent genotype–phenotype correlation studies emphasize that different mutations, even within the same gene, can result in distinct clinical trajectories [8].

Mutations in key genes disrupt essential photoreceptor functions, triggering a cascade of degenerative processes. For example, *RPE65* mutations impair the visual cycle by disrupting the conversion of all-trans-retinol to 11-cis-retinal, a critical process for phototransduction [15]. Similarly, *RPGR* mutations, which primarily affect ciliary transport mechanisms in photoreceptors, contribute to the progressive degeneration of both rods and cones [16]. *USH2A* mutations disrupt the structural organization of retinal photoreceptors, leading to compromised cellular integrity with progressive cell loss [17]. Mutations in *RHO*, which encode the light-sensitive rhodopsin protein in rod cells, lead to protein misfolding, endoplasmic reticulum stress, and finally to increased susceptibility to photoreceptor apoptosis [18].

Collectively, these mutations impair essential photoreceptor functions, leading to metabolic imbalance, oxidative stress, and the activation of proinflammatory and apoptotic pathways, ultimately culminating in photoreceptor cell death.

### 2.2. Mechanisms of Retinal Degeneration

The effects of mutations initiate a cascade of degenerative processes in the retina, which follows a series of interrelated mechanisms primarily driven by photoreceptor cell apoptosis. Rod cells, with their high metabolic demands and oxygen consumption, are especially susceptible to the oxidative stress caused by the production of reactive oxygen species (ROS)—a byproduct of their intense aerobic metabolism [19]. As rod cells degenerate, the oxygen demand in the retina decreases, leading to increased levels of local oxygen, a condition known as retinal hyperoxia. This excess oxygen in the retina promotes the production of ROS, which increases oxidative damage to the remaining photoreceptors and retinal pigment epithelial (RPE) cells [20]. This secondary degeneration of retinal photoreceptors caused by the excessive oxidative stress from initial rod cell death, despite direct genetic defects, has been described as the bystander effect and is a hallmark of RP progression [21].

Inflammatory responses also play a key role in the pathogenesis of RP. The degeneration of photoreceptors triggers microglial activation--the primary immune cells of the retina—which release proinflammatory cytokines, leading to chronic inflammation and exacerbating retinal damage [22]. In addition, dysregulated microglial phagocytic activity has been described, contributing to the excessive clearance of stressed photoreceptors and promoting further neuronal loss. Worsening the degenerative cascade, the accumulation of misfolded proteins due to genetic mutations activates intracellular stress pathways, notably the unfolded protein response (UPR), which further drives photoreceptor apoptosis [18].

### 2.3. Genetic Heterogeneity

The clinical presentation of RP varies significantly based on the underlying genetic mutation and inheritance pattern. Autosomal dominant RP (AdRP), typically associated with *RHO* mutations, generally progresses more slowly than autosomal recessive RP (arRP). The latter results from loss-of-function gene mutations such as *USH2A* and *RPE65* [4]. X-linked RP, often linked to *RPGR* mutations, is one of the most severe forms, with the early onset and rapid progression of the disease [23]. The identification of the gene and its inheritance pattern helps predict the phenotypic expression and prognosis of the disease. Furthermore, it underscores the clinical heterogeneity in patients with RP due to various gene mutations.

Recent studies suggest that modifier genes, epigenetic factors, and environmental influences can further modulate disease severity. For example, patients with identical mutations may have varying degrees of photoreceptor dysfunction, suggesting that additional genetic and non-genetic factors contribute to disease progression [24,25,26].

## 3. Genetic Therapy Strategies

Understanding the complex genetic framework is essential for developing targeted and personalized treatment strategies. In recent decades, advancements in molecular genetics and retinal biology have transformed the therapeutic view for retinitis pigmentosa. As traditional interventions primarily offer symptomatic relief or the subtle preservation of visual function, they fail to address the root genetic causes driving photoreceptor degeneration. With the identification of specific pathogenic mutations and a deeper understanding of disease mechanisms, gene-based therapies have emerged as a promising tool for targeted intervention. These novel strategies aim to slow disease progression and restore visual function by correcting for the underlying genetic defects. We will explore the major categories of genetic therapies currently under investigation or in clinical use for RP.

### 3.1. Gene Replacement Therapy

Gene replacement therapy aims to restore retinal function by delivering copies of genes into retinal cells harboring pathogenic mutations, typically using viral vectors. One of the most notable clinical applications is voretigene neparvovec-rzyl (Luxturna^®^), the first FDA-approved gene therapy for an inherited retinal disease. Luxturna uses an adeno-associated viral (AAV) vector–mediated therapy for *RPE65*-associated retinal dystrophy [27].

In the phase 3 clinical trial, 29 patients with *RPE65*-associated retinal dystrophy received subretinal injections of Luxturna. At 1-year post-treatment, 65% of participants demonstrated clinical improvement in functional vision, as assessed by the multi-luminance mobility test (MLMT), compared to only 10% in the control group. Additionally, full-field light sensitivity threshold (FST) and BCVA revealed improvements with effects sustained through long-term follow-ups, with some seen for up to 4 years. Ultimately, no serious adverse events related to the gene product were reported, confirming a favorable safety profile [27,28]. After Luxturna’s success, multiple ongoing clinical trials have explored gene replacement approaches for other RP-related mutations. For instance, a phase 1/2 in-human clinical trial evaluated an AAV-based vector encoding an optimized *RPGR* gene in patients with X-linked RP. Preliminary results at 6 months demonstrated preserved retinal structure and visual function with no dosage-associated limiting toxicities [29]. These efforts suggest gene replacement therapy as a promising viable treatment option for specific RP subtypes.

Despite these successes, challenges persist in optimizing gene delivery to the retina. The complex structural organization of the retina complicates vector penetration and limits efficient transduction. For example, subretinal injection allows direct delivery to photoreceptors and RPE cells but is invasive and carries the risk of retinal detachment and inflammation. In contrast, despite being less invasive with decreased risks, intravitreal injection often results in low transduction efficiency due to dilution in the vitreous and the potential immune responses within the vitreous [30]. Furthermore, AAV vectors have a limited packaging capacity, restricting their use in diseases caused by mutations in large genes [31]. This capacity represents a significant challenge when targeting genes with large coding sequences, such as *IFT140*, *USH2A*, and *EYS*, all implicated in various forms of RP.

A further challenge in AAV-mediated gene delivery for RP arises from pre-existing immunity. Humans are naturally exposed to wild-type AAV serotypes, particularly AAV2, resulting in neutralizing antibodies that may interfere with vector efficacy. This is especially relevant for intravitreal administration, where the vector is more likely to interact with immune components in the vitreous. In contrast, subretinal delivery offers partial immune privilege by limiting systemic exposure. Nevertheless, serotype selection remains critical. Several ongoing trials utilize engineered or less prevalent serotypes (e.g., AAV8, AAV9) to enhance transduction efficiency and reduce the risk of immune neutralization. Strategies such as immune screening before treatment and immunomodulatory regimens are also under investigation to address this barrier in gene therapy for inherited retinal diseases [31].

In addition to immune neutralization, AAV-based gene therapies have raised safety concerns related to potential liver toxicity, particularly with systemic or high-dose administration. Although ocular gene delivery is largely localized, minimizing systemic exposure, recent reports have identified hepatotoxic effects in some AAV serotypes under different delivery contexts. While such risks are reduced in the subretinal delivery used in RP trials, caution remains warranted, especially as novel serotypes and delivery routes are explored [31].

Multi-vector recombination strategies have been studied to address the limited packaging capacity. For example, Datta and co-workers implemented a recombination system using the CRE-lox system to deliver the large *IFT140* gene [32]. The approach utilized up to four AAV vectors, each encoding fragments of the therapeutic gene. The strategy successfully reconstituted the full-length *ITF140* in cultured mammalian cells and mouse retinas. Importantly, when dual AAV vectors delivering the *IFT140* gene were administered in the sub-retinal space in a conditional knockout mouse model, the therapy significantly preserved the photoreceptor structure, maintained retinal function (measured via ERG response), and prevented retinal degeneration [32]. While these findings demonstrate promising preclinical efficacy, the Cre-lox system presents translational challenges for human therapy. These include concerns about recombination efficiency in human retinal tissue, vector packaging complexity, and regulatory hurdles related to the co-administration of multiple viral vectors. Additional studies are needed to optimize recombination fidelity, assess immunogenicity, and evaluate the safety profile in large-animal models before considering clinical application.

Another innovative approach involves the use of split intein-mediated protein trans-splicing. This method allows for the delivery of large genes by splitting them into smaller fragments that can be packaged into separate AAV vectors. For example, Tornabene and co-workers demonstrated the successful expression of full-length *ABCA4* and *CEP290* in mouse and pig retinas [33]. Despite these challenges, ongoing innovations in vector design, delivery techniques, and immune modulation are steadily improving the safety and effectiveness of retinal gene therapy.

### 3.2. Gene Editing Approaches

Gene editing approaches are promising for treating patients with RP by correcting deleterious mutations at the DNA level [34]. Among these, the Clustered Regularly Interspaced Short Palindromic Repeats/CRISPR-associated protein 9 (CRISPR/Cas9) is a gene-editing system derived from bacterial adaptive immunity that enables site-specific DNA cleavage and repair through the guidance of RNA molecules [35]. This technology allows one to make precise modifications to the genome, restoring normal gene function and halting disease progression [34]. A landmark example is EDIT-101, a CRISPR/Cas9 gene-editing complex targeting the *CEP290* gene. In the phase 1/2 BRILLIANCE clinical trial, Pierce and co-workers reported that 43% of patients who received EDIT-101 demonstrated a visually meaningful improvement in retinal sensitivity, with additional gains observed in BCVA and quality-of-life measures [36]. Preclinical studies have shown that CRISPR/Cas9 can effectively target and correct gene mutations such as *RPGR*, leading to photoreceptor preservation and functional improvement in mouse models. Notably, no detectable off-target editing effects, where unintended DNA regions may be altered, were reported for up to 12 months following treatment [37].

However, despite these promising results in gene editing, several ethical considerations should be addressed. The potential unintended genetic modifications raise safety concerns. Also, long-term monitoring is essential to ensure that gene editing does not introduce further deleterious mutations or trigger immune responses [38].

### 3.3. RNA-Based Therapies

RNA-based therapies offer alternative strategies for treating patients with RP by targeting mutant mRNA transcripts. Antisense oligonucleotides (AONs) are short, single-stranded DNA or RNA molecules that specifically bind to complementary sequences on target pre-mRNA. Hence, AONs can modulate RNA processing in several ways, notably by blocking access to splice sites. In the context of RP, AONs are particularly useful in correcting aberrant splicing events caused by intronic or exomic mutations, ultimately restoring the open reading frame and enabling the translation of a functional protein [39].

For example, Grainok and co-workers described an AON-based exon-skipping strategy to treat RP, specifically RP11, which truncated the mutations of the pre-mRNA processing factor 31 (*PRPF31*) gene [40]. The study demonstrated that AONs could induce the selective skipping of the mutated exon, restoring the gene’s open reading frame and enhancing the production of functional PRPF31 protein [40]. Similarly, research by Slijkerman and co-workers explored the use of AONs to address a deep-intronic mutation in the *USH2A* gene, which leads to the inclusion of a pseudoexon and results in a truncated, non-functional usherin protein [41]. Their work demonstrated that AON-mediated splice correction can prevent the incorporation of the pseudoexon, thereby restoring regular usherin protein expression and function [41]. Both these studies highlight the therapeutic potential of AONs in RP by precisely manipulating mRNA splicing events to overcome the effects of deleterious mutations.

While complementing the splice-modifying capacities of AONs, RNA interference (RNAi) offers a distinct mechanism of action by selectively silencing mutant gene transcripts. This process is mediated by small interfering RNAs (siRNAs) or short hairpin RNAs (shRNAs), which are designed to specifically bind complementary sequences within the target mRNA, leading to its cleavage and preventing translation into dysfunctional proteins. Specifically, Guzman-Aranguez and co-workers reviewed several RNAi-based strategies targeting retinal diseases, highlighting the therapeutic potential of siRNAs in RP by silencing pathogenic rhodopsin transcripts [42]. O’Reilly and co-workers demonstrated the suppression of mutant rhodopsin (*RHO*) transcripts via the application of RNAi in patients with autosomal dominant RP. The study showed that RNAi can have up to a 90% suppression of *RHO* expression in photoreceptors using AAV vectors for delivery [43]. Therefore, these studies underscore the potential of RNA-based therapies in RP, offering a targeted approach to mitigate photoreceptor degeneration and preserve visual function.

While both gene editing and RNA-based therapies offer targeted treatment strategies for RP, they differ significantly in mechanism, durability, and clinical considerations. Gene editing approaches such as CRISPR/Cas9 aim to permanently correct pathogenic DNA mutations through a single intervention, potentially offering long-term or curative benefit. However, safety concerns, particularly related to off-target editing, immunogenicity, and long-term genomic stability, require ongoing monitoring and ethical caution. In contrast, RNA-based therapies like AONs and RNAi modulate gene expression at the transcript level and are generally well tolerated. Their transient nature typically necessitates repeated administration to maintain efficacy but reduces the risk of permanent genomic alteration. From a cost perspective, RNA-based strategies may offer more accessible short-term interventions, whereas gene editing, although more resource-intensive, holds promise for durable, mutation-specific treatment. Together, these approaches represent complementary strategies in the evolving landscape of personalized retinal therapeutics.

### 3.4. Optogenetic Therapy

In contrast to therapies that aim to correct or silence genetic defects, optogenetic therapy offers a novel approach by re-engineering light sensitivity in retinal cells downstream of the photoreceptors. This modality is used for patients with advanced RP, particularly those with extensive photoreceptor loss [44]. This strategy involves introducing genes that encode light-sensitive proteins—such as channel-rhodopsins—into surviving inner retinal neurons, including bipolar or ganglion cells, to confer light responsiveness in the absence of functional photoreceptors [45]. By reactivating these downstream neurons, optogenetics bypasses damaged photoreceptors and creates a new photosensitive pathway [46].

In a clinical case, Sahel and colleagues reported partial visual recovery in a blind RP patient using an optogenetic construct (ChrimsonR) delivered via intravitreal injection, combined with engineered goggles that amplified and converted light into specific wavelengths, demonstrating proof-of-concept for human use [46]. Notably, no reported adverse ocular events over 84 weeks of follow-up were described. Similarly, Lindner and co-workers reviewed multiple preclinical studies where optogenetic tools successfully activated retinal ganglion cells in RP animal models, restoring light responses and allowing basic visual behaviors [46].

The RESTORE trial, a Phase 2b randomized, double-masked, sham-controlled study conducted by Nanoscope Therapeutics, evaluated the efficacy of MCO-010, an ambient light-activatable multi-characteristic opsin (MCO) gene therapy for patients with advanced retinitis pigmentosa. The therapy, delivered via intravitreal injection, demonstrated statistically significant improvements in visual function in preclinical models of retinitis pigmentosa. Treated mice exhibited enhanced optomotor responses, faster spatial navigation in visually guided water-maze tests, and improved visually evoked potentials compared to the controls. These functional gains persisted for up to 26 weeks post-injection. Importantly, MCO-010 was well tolerated, with no evidence of ocular toxicity, phototoxicity, or systemic immunogenicity. These results underscore the potential of optogenetic therapy as a gene-agnostic treatment strategy for patients with late-stage RP [47].

While promising, optogenetic therapy is currently limited by the need for external light amplification devices, restricted spatial resolution, and dependence on viable inner retinal circuits. Nonetheless, this approach holds significant potential for patients in the late stages of RP, where traditional photoreceptor-targeted therapies are no longer viable.

Table 1 summarizes the primary gene-based therapeutic strategies currently explored in retinitis pigmentosa, detailing their mechanisms of action, delivery methods, outcomes, and limitations in clinical and preclinical settings.

## 4. Challenges in Genetic Therapy

### 4.1. Delivery Mechanisms

Despite genetic therapies’ promising potential for RP, several challenges must be addressed to ensure their long-term safety, efficacy, and accessibility. One primary limitation involves the delivery of therapeutic genes to retinal cells.

Viral vectors, most commonly adeno-associated viruses (AAVs), are the leading medium for gene delivery due to their natural affinity for retinal cells, known as tropism, and safety profile [27]. However, AAVs have a limited carrying capacity (<5 kb), making them unsuitable for larger genes. This constraint renders AAV vectors unsuitable for delivering large genes, including *USH2A*, *EYS*, or *ABCA4*, which exceed this size threshold [33,48]. For example, Trapani and co-workers emphasized that approximately one-third of genes implicated in inherited retinal diseases exceed the natural capacity of AAV vectors, presenting an obstacle to therapeutic development [49]. Their review study highlighted that an attempt to compress larger cDNAs into a single AAV resulted in unstable products, leading to reduced transduction efficiency and inconsistent therapeutic expression. Similarly, Toms and co-workers noted that the full-length *USH2A* was three times the supposedly accommodated size, hence proposing the necessity for alternative strategies for effective gene delivery [48].

Physical and anatomical barriers within the eye also significantly limit AAV-mediated transduction, particularly when vectors are delivered intravitreally. Dalkara and co-workers demonstrated that the inner limiting membrane (ILM) acts as a barrier to AAV penetration, limiting gene expression in the inner retina [50]. Specifically, the study found that the co-administration of a protease to digest the ILM enabled the specific AAV serotype to transduce multiple retinal layers, an effect not previously achieved via intravitreal delivery. Subretinal injection, though effective in bypassing the ILM, is invasive and carries surgical risks such as retinal detachments [51]. In their study involving non-human primates, Gamlin and co-workers observed procedures leading to localized retinal damage. They proposed that the meticulous control during injected delivery is required to prevent iatrogenic trauma [51]. Therefore, modern subretinal injections are now guided by OCT imaging to improve safety and accuracy, allowing clinicians to monitor needle positioning during the procedure.

Beyond delivery efficiency, safety concerns such as immune responses to the viral capsid or gene product pose further obstacles. Bucher and co-workers demonstrated that these immune responses could lead to inflammation and reduced therapeutic durability, particularly in the context of adeno-associated virus (AAV) vectors used for gene therapy [31]. They showed that AAV vectors can activate Toll-like receptors (TLR), resulting in the release of inflammatory cytokines and interferons. Additionally, AAV vectors can induce capsid-specific and transgene-specific T-cell responses and anti-AAV antibodies, which limit therapeutic effects by destroying targeted cells [31].

### 4.2. Long-Term Efficacy and Safety

Another consideration is the sustainability of therapeutic effects over time. Although early clinical trials have shown encouraging results, long-term data on gene therapy durability is still limited. Maguire and co-workers reported that the therapeutic effects of voretigene neparvovec-rzyl (Luxturna^®^) were maintained for up to four years in patients with *RPE65*-associated retinal dystrophy [52]. However, Chao and co-workers also noted instances of visual function decline in some patients [53]. Concerns remain regarding gene expression stability, progressive retinal remodeling, and the potential for delayed adverse effects. As such, long-term follow-up and robust post-treatment monitoring will be essential to ensure efficacy and safety across diverse patient populations.

### 4.3. Personalized Medicine and Genetic Variability

Finally, the considerable genetic and phenotypic heterogeneity of RP presents challenges for personalized treatment strategies. With over 3000 mutations identified across more than 70 genes, therapies must be tailored to individual genotypes, requiring accessible and comprehensive genetic testing alongside mutation-specific delivery platforms. As highlighted by Dias and co-workers, this genetic heterogeneity necessitates the development of genotype-specific therapies and broad access to diagnostic testing to improve patient selection and clinical outcomes [12]. Similarly, Nguyen and co-workers emphasized the essential role of genetic testing not only for accurate diagnosis but also for determining the eligibility for mutation-specific therapies and advancing enrollment in appropriate clinical trials [54].

Gene replacement therapy is considered most effective in cases where the underlying mutation results in a loss of gene function and the therapeutic gene is small enough to fit within the packaging limits of vectors. As noted by Arbabi and co-workers, this approach is particularly applicable for patients with autosomal recessive RP, where biallelic loss-of-function mutations result in the absence of functional proteins [55]. Similarly, Dias and co-workers highlight that gene augmentation strategies are appropriate when the defective gene can be replaced with a functional copy [12]. However, both studies underscore the limitation regarding the packaging capacity of specific vectors such as AAVs.

In contrast, RNA-based therapies or gene editing may be more appropriate, especially when dominant-negative or splicing mutations are involved. For example, antisense oligonucleotides can modulate splicing defects, while gene editing technologies like CRISPR/Cas9 can correct specific mutations at the DNA level [40,56]. Therefore, accurate genetic diagnosis is essential to determine eligibility for targeted clinical trials or mutation-specific interventions.

Moreover, treatment response can vary significantly based on disease stage, retinal health, and individual genetic modifiers, even among patients with the same mutation [8,24,57]. These facts highlight the importance of developing therapeutic platforms that can accommodate interindividual variability, such as genetic testing, clinical profiling, and biomarker identification. Personalized approaches will be critical in advancing equitable and effective care for individuals living with RP.

Table 2 summarizes the major challenges encountered in the application of genetic therapy for RP, highlighting both the limitations and emerging solutions.

## 5. Recent Advances and Clinical Trials

### 5.1. Overview of Key Clinical Trials

Currently, gene therapy is considered a promising treatment option for patients with RP, particularly those with biallelic *RPE65* mutations. The approval of Luxturna marked a milestone in ocular gene therapy, offering the first FDA-approved gene therapy for inherited retinal disease. In a phase 3 clinical trial, Russell et al. demonstrated significant improvements in functional vision, with 72% of patients (21 of 29) achieving successful performance on the MLMT at the lowest light level (1 lux) one-year post-treatment, compared to none achieved at baseline [28]. These results were supported by Maguire and co-workers, who demonstrated gains in light sensitivity, with mean improvements in full-field light sensitivity threshold (FST) of −2.04 log10 (cd·s/m) sustained through 3 years of follow-up [58], that is, more than 100-fold enhancement in light sensitivity.

Furthermore, visual field expansion and stability in visual acuity were also observed. For example, at 4-year follow-ups, Maguire and co-workers described patients in the original intervention group with a mean increase of 197.7 degrees in Goldmann visual field and maintained near-baseline visual acuity [58]. Similarly, in another study by Maguire, the group confirmed the long-term durability of these effects, noting that the gains in FST and MLMT performance were preserved for up to 4 years, with no serious adverse events reported, affirming the favorable safety profile of Luxturna^®^ [52]. In some instances, adverse effects were mild and mostly attributed to the subretinal surgical procedure rather than the vector itself [52]. Hence, these results from clinical trials provide evidence that gene replacement therapy with voretigene neparvovec leads to sustained improvements in vision-related function.

Beyond *RPE65*, gene therapy trials are now expanding to address other RP genotypes. A phase I/II clinical trial (XIRIUS) targeting *RPGR*-related X-linked RP demonstrated encouraging early results. Cehajic-Kapetanovic and co-workers reported that patients receiving mid-range doses of an AAV vector to deliver codon optimized version of *RPGR* exhibited gains in retinal sensitivity and the partial reversal of visual field loss as early as 1 month, with sustained effects at 6 months [29]. However, in higher dose cohorts, cases of mild subretinal inflammation were reported.

Additionally, Pierce and co-workers evaluated EDIT-101, the first in vivo CRISPR/Cas9-based gene-editing therapy, in patients with *CEP290*-associated Leber Congenital Amaurosis (LCA10) [36]. Although LCA10 is not a form of RP, the BRILLIANCE trial represents a significant proof-of-concept for CRISPR in the retina. The study found that 64% of patients had a clinically meaningful improvement in at least one visual outcome (e.g., FST, best-corrected visual acuity, or mobility test), with no dose-limiting toxicities or serious adverse events attributed to the treatment [36]. Although still in early phases, preclinical studies have demonstrated that gene editing approaches targeting *RPGR*, including CRISPR-mediated frame restoration in ORF15, can successfully rescue photoreceptor structure and function in RP models [29,59,60]. These findings collectively underscore the therapeutic potential of gene editing platforms in inherited retinal diseases and provide a way for broader applications across RP genotypes.

Other ongoing trials are exploring gene editing technologies, such as CRISPR/Cas9, to correct mutations in genes like *CEP290* and *RPGR*, with early-stage research showing promising results in preclinical and clinical settings. For instance, a Phase 1/2 clinical trial (BRILLIANCE) is evaluating the safety and efficacy of EDIT-101, a CRISPR/Cas9-based therapy targeting the *CEP290* mutation associated with Leber Congenital Amaurosis 10 (LCA10). Although LCA10 is not a subtype of RP, the trial is significant as it represents the first clinical application of CRISPR in the retina, demonstrating in vivo gene editing. Initial findings have demonstrated favorable safety outcomes and preliminary evidence of improvements in full-field stimulus testing (FST) and best-corrected visual acuity (BCVA) in some treated patients [36].

### 5.2. AI-Driven Precision Medicine and Retinal Prosthetics

The integration of artificial intelligence (AI) and machine learning in RP research has the potential to revolutionize the identification of candidate genes and the optimization of therapies. These technologies enable the analysis of large-scale genetic and clinical datasets, allowing for the classification of inherited retinal diseases (IRDs), the prediction of causative genes, and disease prognosis. A study by Esteban-Medina and co-workers used machine learning to generate a mechanistic functional map of RP, identifying 226 functional circuits and predicting 109 targets of approved drugs with potential effects [61]. Some of these targets were validated in a murine model, highlighting the potential of AI-driven approaches for drug repurposing and novel therapeutic discovery [61]. Additionally, Gomes and Ashley highlighted the application of AI in molecular medicine, emphasizing its role in variant prioritization for rare diseases [62]. This ability is crucial for RP, where over 70 genes have been implicated. Fujinami-Yokokawa and co-workers demonstrated that deep learning models trained on fundus photographs and fundus autofluorescence images could predict causative IRD genes such as *EYS*, *ABCA4*, and *RP1L1* with test accuracies of up to 88.2% and specificities exceeding 95%, illustrating AI’s potential to enhance non-invasive diagnostic precision in RP [63]. Similarly, Issa and co-workers noted that AI models applied to retinal imaging were able to discriminate RP images from normal images with a value of area under the receiver operating curve (AUROC) of 96.74% [64].

By leveraging machine learning algorithms, researchers and clinicians can uncover novel genetic associations and lead treatment strategies tailored to individual genetic profiles. The AI-driven methodologies are pivotal in advancing precision medicine for RP, enabling tailored interventions based on individual genetic profiles and optimizing patient outcomes.

Concurrently, advances in retinal prosthetics are expanding the treatment landscape for blind patients with RP. Retinal prosthetic devices such as epiretinal and subretinal implants aim to restore partial vision by stimulating surviving retinal neurons [65]. Clinical trials have demonstrated their potential in restoring visual perception to profoundly blind patients with RP. For example, Ho and co-workers underwent the extensive clinical evaluation of the Argus II Retinal Prothesis System, an epiretinal device [66]. The study involving 30 subjects reported an acceptable safety profile and functional benefit, with the longest implant duration reportedly reaching 7.2 years [66].

### 5.3. Cell-Based Therapies

Cell-based therapies may be used for RP by targeting the underlying degeneration of photoreceptors and retinal pigment epithelium (RPE) cells. These therapies aim to replace damaged retinal cells through the transplantation of stem cell-derived photoreceptors or RPE cells, potentially restoring retinal structure and function.

Preclinical studies have demonstrated that retinal progenitor cells (RPCs) can survive, integrate into the host retina, and promote photoreceptor preservation in degenerative models. Wang and co-workers reported that transplanted RPCs in rodent models of RP led to improvements in visual function and the structural integrity of the outer retina [67]. Early-phase clinical trials have also provided encouraging results. In a systematic review and meta-analysis, Chen and co-workers analyzed 21 prospective studies involving 496 eyes and found that 49% of RP patients experienced an improvement in best-corrected visual acuity (BCVA) at 6 months, although long-term benefits beyond 12 months were less significant [68]. The analysis further highlighted that suprachoroidal space injection showed the most significant BCVA improvement, suggesting it as a delivery route. Additionally, Liu and co-workers demonstrated the long-term safety and feasibility of human fetal-derived RPC transplantation in RP patients, with five out of eight patients showing improved visual acuity and three exhibiting enhanced retinal sensitivity as measured by pupillary light reflex within the first 6 months post treatment, without evidence of immune rejection or tumor formation over a two-year follow-up period [69]. These findings underscore the regenerative potential and safety profile of stem cell-based strategies in inherited retinal degenerations.

However, these technologies face several challenges. For retinal prostheses, limitations include low spatial resolution, complex surgical implantation, and limited patient adaptability. In stem cell therapies, concerns persist regarding long-term graft survival, immune rejection, and the theoretical risk of tumorigenesis. These factors highlight the need for the continued long-term evaluation of safety and efficacy in clinical trials.

Table 3 summarizes key clinical trials and emerging technological strategies in the treatment of RP, providing an overview of the therapeutic indications, major findings, and translational limitations across gene therapy, gene editing, artificial intelligence, and regenerative platforms.

## 6. Future Directions and Perspectives

As the field of RP therapy continues to evolve, future directions are increasingly centered on overcoming the current limitations of gene delivery and maximizing therapeutic efficacy through multimodal approaches. To address these gaps, ongoing research is exploring next-generation delivery systems, including advanced viral vectors, nanoparticles, and non-viral platforms, aimed at improving safety, precision, and accessibility. In parallel, the integration of gene therapy with complementary modalities such as stem cell transplantation and retinal prosthetics is gaining attention as a strategy to enhance outcomes and expand the therapeutic options for individuals with advanced or genetically complex diseases.

### 6.1. Improving Delivery Systems

Effective delivery of therapeutic agents to retinal cells is crucial for the success of gene therapies in RP. Traditional methods, such as subretinal injections using AAV vectors, have shown promise but come with limitations, including surgical risks and limited retinal coverage. Recent innovations that aim to address these challenges include intravitreal AAV vector delivery, nanoparticle technology, and non-viral delivery systems.

ViGeneron has recently launched a clinical trial utilizing their proprietary vgAAV vector for the intravitreal delivery of the *CNGA1* gene, targeting the RP caused by *CNGA1* mutations. Prior studies have demonstrated the potential of variant AAV capsids for efficient intravitreal gene delivery to photoreceptors, crucial for treating inherited retinal diseases. For example, Pavlou and coworkers reported the development of engineered AAV vectors that enable the efficient targeting of photoreceptors via intravitreal administration [70]. In different animal models, the novel variants showed an up to 100-fold higher photoreceptor transduction efficiency compared to the unmodified AAV2 vector—a version widely used in early gene therapy research and clinical trials [70].

Additionally, He and co-workers identified a novel AAV2 capsid variant that demonstrated key improvements in treated mice, supporting its clinical potential [71]. Specifically, the intravitreal administration of the variant capsid carrying the affected gene led to significantly restored visual function, as evidenced by improved pupillary light reflex and behavioral performance in light/dark box and visual cliff tests. Similarly, through histological analysis, the authors revealed that treated mice had a preserved outer nuclear layer (ONL) thickness and significantly increased expression of the affected gene product compared to those receiving standard AAV2 vectors [71]. These two examples further support the current direction towards improving vector design.

Nanoparticle-based delivery systems, such as mesoporous silica-based nanoparticles, polymeric nanoparticles, lipid nanoparticles, and other non-viral vectors, are emerging as a promising alternative to traditional viral vectors for gene therapy in RP. These offer advantages over traditional viral vectors, such as higher safety profiles, low immunogenicity, and the capacity to deliver larger genetic payloads. In a study by Valdés-Sanchez and co-workers, mesoporous silica nanoparticles (MSNs) mediated the successful delivery of therapeutic genes to retinal cells in vivo without disrupting retinal morphology or function, as confirmed through multimodal imaging and electroretinographic studies [72]. Similarly, Maya-Vetencourt and co-workers demonstrated that conjugated polymer nanoparticles such as poly(3-hexylthiophene) (P3HT) could restore visual function in RP rat models, showing widespread the subretinal distribution of P3HT nanoparticles and long-term efficacy without triggering retinal inflammation or immune response [73]. Further supporting its potential, Francia and co-workers demonstrated that P3HT nanoparticles restored cortical visual responses in advanced-stage RP rats, even after photoreceptor degeneration [74]. Collectively, these findings indicate the biocompatibility and short-term therapeutic efficacy of non-traditional delivery systems.

### 6.2. Combination Therapies

Combining gene therapy with other treatment modalities, such as stem cell therapy and retinal implants, represents a promising future direction for enhancing therapeutic outcomes in RP. By integrating these approaches, researchers aim to simultaneously target both genetic defects and cellular degeneration. Several studies have explored the feasibility and efficacy of these combination therapies. MacLaren et al. emphasized that gene and cell-based therapies should be viewed as complementary strategies, with gene therapy offering the greatest benefit in early stages where viable photoreceptors are still present, and stem cell transplantation being more applicable in the advanced stages marked by widespread retinal cell degeneration [13].

Furthermore, Sahel and co-workers provided compelling clinical evidence supporting the integration of optogenetic therapy and retinal prosthetics in a first reported case; by delivering optogenetic gene constructs intravitreally and combining this intervention with light-stimulating goggles, they achieved the partial restoration of visual function in a blind RP patient, underscoring the potential of combined genetic-device interventions [46]. Collectively, these findings emphasize the substantial potential of multimodal therapeutic strategies to address the complex pathology of RP and enhance patient outcomes.

### 6.3. Regulatory and Ethical Issues

As therapeutic strategies for RP rapidly advance, navigating regulatory pathways and addressing ethical considerations will be critical to translating novel gene therapies into clinical practice. Gene therapies, including advanced vectors and combinational approaches, must undergo rigorous assessment by regulatory authorities such as the U.S. Food and Drug Administration (FDA) and the European Medicines Agency (EMA). The regulatory approval process involves multiple phases of clinical trials designed to thoroughly evaluate safety, efficacy, and long-term outcomes, often requiring extensive follow-up data to substantiate therapeutic benefits and identify potential risks [75]. The landmark approval of Luxturna^®^ (voretigene neparvovec-rzyl), an AAV-based gene therapy for inherited retinal dystrophy caused by *RPE65* mutations, exemplifies the complexity and rigor of these processes and has paved the way for future RP therapies [28].

In parallel with regulatory challenges, ethical concerns surrounding gene therapies for RP must also be carefully considered. Genetic manipulation, particularly techniques involving gene editing or germline modifications, raises significant ethical questions related to potential off-target effects, long-term safety, and the implications of irreversible genomic alterations [76]. Moreover, equitable access to emerging gene therapies remains a significant concern due to their typically high costs, potentially limiting availability to select populations and exacerbating existing healthcare disparities [77]. Ensuring fair distribution and access to these therapies will necessitate ongoing dialogue between researchers, clinicians, policymakers, and patient advocacy groups.

As treatments for patients with RP evolve toward personalized, multimodal interventions, addressing these regulatory and ethical challenges will be crucial to ensuring safe, effective, and accessible therapies for patients worldwide.

### 6.4. The Path Forward for RP Patients

Advancements in the molecular understanding of RP, along with significant progress in gene therapy platforms, are guiding the way for more personalized treatment strategies for patients with diverse genetic subtypes. Due to RP caused by mutations in over 80 different genes, its genetic heterogeneity poses a major therapeutic challenge. However, recent studies emphasize that expanding genetic screening and variant classification may facilitate the development of mutation-specific therapies [78].

Promising advancements are emerging for specific subtypes such as *RPE65*-, *RPGR*-, and *USH2A*-associated RP. These targeted gene therapy approaches are currently under investigation in clinical trials, with the potential to advance towards further stages of development. Furthermore, innovative non-viral delivery systems, nucleic acid base editing, and CRISPR/Cas9 gene-editing technologies are being explored to expand the spectrum of treatable mutations and address the limitations associated with vector capacity and delivery efficiency [12,78]. These advancements, in addition to the increasing efforts in clinical trial development, suggest that gene therapy may soon evolve from experimental intervention to a standard therapeutic modality for specific genetic subsets of RP.

## 7. Conclusions

Genetic therapy has reshaped the treatment landscape of RP, shifting from symptomatic management toward targeted molecular interventions. Advances in gene replacement (e.g., Luxturna^®^ for *RPE65*), gene editing (e.g., EDIT-101 for *CEP290*), RNA-based modulation, and optogenetic therapy have demonstrated clinical and preclinical success across various RP genotypes. Additionally, novel delivery strategies, including engineered AAV serotypes and nanoparticle platforms, are helping overcome packaging and transduction limitations, expanding the range of targetable mutations, including those previously considered untreatable due to gene size.

Despite these successes, the therapeutic potential of genetic interventions in RP will depend on overcoming several key challenges, including improving delivery efficiency, addressing immune responses, and ensuring long-term safety and efficacy. Given RP’s considerable genetic and phenotypic heterogeneity, precision medicine guided by early genetic diagnosis is essential to identify patients to mutation-specific therapies and implement personalized treatment strategies.

Looking ahead, the translation of experimental therapies into standard clinical practice will require rigorous long-term safety monitoring, scalable delivery systems, and equitable access frameworks. Ultimately, these innovative approaches offer hope to transform the prognosis of patients with RP from progressive vision loss to a future where disease stabilization, and even functional recovery, becomes a realistic goal.

## Figures and Tables

**Table 1:** Summary of gene-based therapeutic strategies in RP.

Therapy Strategy	Mechanism/Target	Delivery Method	Recent Outcomes	Limitations
Gene Replacement Therapy	AAV-delivered gene copies to restore function (e.g., *RPE65*, *RPGR*); dual/multi-vector recombination for large genes (e.g., *IFT140*)	Subretinal injection (AAV2); dual vectors for large genes	Luxturna^®^: 65% improved MLMT at 1 year; *RPGR* trials show structural/functional preservation; *IFT140* dual AAV preserved function in mice	AAV packaging limits; subretinal surgery risks (detachment, inflammation)
Gene Editing (CRISPR-Cas9)	DNA-level correction (e.g., *CEP290*, *RPGR*) via RNA-guided Cas9 to restore gene function	Subretinal delivery of CRISPR complexes via viral or non-viral systems	EDIT-101: 43% showed improved retinal sensitivity; RPGR editing in mice preserved photoreceptors without off-target effects	Risk of off-target edits; ethical/safety concerns; need for long-term monitoring
RNA-Based Therapies (AON, RNAi)	AONs modify splicing (e.g., *PRPF31*, *USH2A*); siRNA/shRNA silence toxic mRNA (e.g., *RHO*)	Intravitreal injection of synthetic oligos or viral-delivered RNA tools	AONs corrected splicing in *PRPF31* and *USH2A* models; RNAi achieved 90% *RHO* transcript suppression in photoreceptors	Transient effects; limited delivery efficiency; immune response potential
Optogenetic Therapy	Introduces light-sensitive opsins (e.g., ChrimsonR, MCO-010) into bipolar/ganglion cells to bypass lost photoreceptors	Intravitreal injection of gene constructs (no viable photoreceptors needed)	RESTORE trial: mice showed improved optomotor and water-maze behavior; human case regained partial vision using ChrimsonR with engineered goggles; well tolerated	Low spatial resolution; dependent on assistive devices; requires intact downstream retinal circuitry

**Table 2:** Challenges in genetic therapy for RP.

Challenge	Advantage/Opportunity	Limitation	Proposed Solutions
Delivery Barriers	Subretinal injection allows precise targeting; capsid engineering improves vector spread	Invasive procedure; risk of detachment or inflammation; intravitreal injection has low efficiency	Use of suprachoroidal delivery, capsid optimization, and hybrid delivery systems
Vector Capacity Limitations	Gene editing and RNA-based therapies allow payload minimization	AAVs cannot carry large genes (e.g., *USH2A*, *ABCA4*)	Dual/multi-vector approaches, split-intein recombination, or alternative vectors
Immune Responses	Novel serotypes and immune modulation strategies enhance tolerability	Vector-induced immune activation may limit expression or cause inflammation	Pre-treatment with immunosuppressants, vector engineering, and monitoring biomarkers
Gene and Mutation Specificity	Precision medicine enables mutation-matched therapy	Rare variants may be untargetable or underrepresented in clinical research	Integrate gene-agnostic methods (e.g., optogenetics); develop mutation-agnostic delivery platforms
Long-Term Expression and Safety	Episomal vectors reduce genomic integration; regulatory elements can fine-tune expression	Concerns about sustained expression, gene silencing, or unforeseen effects	Use of biodegradable vectors, inducible promoters, and long-term clinical surveillance
Ethical and Regulatory Concerns	Global consensus growing around clinical gene therapy governance	Issues around germline editing, patient autonomy, and equitable access	Clear ethical oversight, patient engagement, transparent consent processes, and international policy alignment

**Table 3:** Summary of notable clinical trials and emerging technologies in RP.

Intervention/Technology	Target/Indication	Key Clinical Findings	Limitations/Considerations
Luxturna^®^ (voretigene neparvovec)	*RPE65*-associated RP	72% MLMT success at 1 lux; FST gain −2.04 log10 cd·s/m; sustained visual field and acuity improvements for 4+ years	Requires subretinal surgery; gene-specific; not generalizable to all RP genotypes
RPGR Gene Therapy (XIRIUS trial)	X-linked RP (*RPGR* mutations)	Improved retinal sensitivity and partial visual field reversal in mid-dose groups; benefits observed as early as 1 month, sustained at 6 months	Mild subretinal inflammation at higher doses; limited to *RPGR* mutations
EDIT-101 (BRILLIANCE trial)	*CEP290* mutation (LCA10—CRISPR/Cas9 gene editing)	64% had improvements in ≥1 visual function outcome (FST, BCVA, mobility); no serious adverse events	Still in early phases; LCA10 not classified as RP but establishes retinal CRISPR precedent
AI Applications in RP	Gene identification, diagnosis, prognosis	Predicted causative genes (e.g., *EYS*, *RP1L1*) with 88.2% accuracy; AUROC 96.74% in retinal image-based classification	Not a therapy; dependent on algorithm quality, training data, and clinical integration
Argus II Retinal Prosthesis System	Advanced RP with profound vision loss	Clinical safety and functional benefit in 30 patients; device longevity up to 7.2 years	Low visual resolution; surgical implantation complexity; limited adaptability
Stem Cell Therapy (e.g., RPCs via Suprachoroidal Delivery)	RP with photoreceptor/RPE degeneration	49% of eyes showed BCVA improvement at 6 months (n = 496); best results with suprachoroidal delivery; no rejection/tumors in long-term follow-up trials	Outcomes vary by protocol; long-term efficacy unclear; risk of immune rejection and graft survival limitations

## Data Availability

No new data were created or analyzed during this study. Data sharing is not applicable to this article.

## Abbreviations

The following abbreviations are used in this manuscript:

AAV	Adeno-Associated Virus
AI	Artificial Intelligence
AON	Antisense Oligonucleotide
BCVA	Best-Corrected Visual Acuity
CEP290	Centrosomal Protein of 290 kDa
CRISPR	Clustered Regularly Interspaced Short Palindromic Repeats
ERGs	Electroretinograms
FST	Full-Field Light Sensitivity Threshold
ILM	Inner Limiting Membrane
IRDs	Inherited Retinal Diseases
LCA	Leber Congenital Amaurosis
MLMT	Multi-Luminance Mobility Test
MSNs	Mesoporous Silica Nanoparticles
ONL	Outer Nuclear Layer
P3HT	Poly(3-Hexylthiophene)
PRPF31	Pre-mRNA Processing Factor 31
RHO	Rhodopsin
RP	Retinitis Pigmentosa
RPGR	Retinitis Pigmentosa GTPase Regulator
RPE	Retinal Pigment Epithelium
siRNA	Small Interfering RNA
shRNA	Short Hairpin RNA
UPR	Unfolded Protein Response
USH2A	Usher Syndrome Type 2A
